# Efficacy of low-level laser therapy in pain relief during orthodontic treatment: Protocol for a systematic review and meta-analysis

**DOI:** 10.1371/journal.pone.0352702

**Published:** 2026-07-09

**Authors:** Cintia Ronchi Lemos, Daniela Bianchini Orlandi, Anna Carolina Pereira Thimoteo, Helena Polmann, Patrícia Pauletto, Cristine Miron Stefani, Eliete Neves Silva Guerra, Carlos Flores-Mir, Graziela De Luca Canto

**Affiliations:** 1 Brazilian Centre for Evidence-Based Research (COBE), Brazil; 2 Federal University of Santa Catarina (UFSC), Department of Dentistry, Florianópolis, Brazil; 3 School of Dentistry, Universidad De Las Americas (UDLA), Quito, Ecuador; 4 University of Brasília (UnB), School of Health Sciences, Brasília, Brazil; 5 Mike Petryk School of Dentistry, University of Alberta, Edmonton, Canada; 6 Postgraduate Program in Evidence-Based Health, Federal University of São Paulo, São Paulo, Brazil; University of Zurich, SWITZERLAND

## Abstract

**Introduction:**

Patient adherence to orthodontic treatment depends on comfort, absence of pain, and improved quality of life. Pain and discomfort from procedures like interproximal separation or appliance activation are often underestimated by professionals. Low-level laser therapy, with minimal side effects, shows potential for pain relief in orthodontics, though its efficacy remains uncertain.

**Objectives:**

To investigate the efficacy of low-level laser therapy in controlling pain in orthodontic patients.

**Methods:**

A systematic review of randomized controlled trials with orthodontic patients assessing low-level laser therapy will be done. Studies with specific treatments, such as hybrid orthodontics with skeletal anchorage or intermaxillary elastics, will be excluded. The intervention will compare low-level laser therapy alone versus sham therapy, no treatment, or other interventions. No restrictions will apply regarding publication date, language, or patient age. Primary outcomes will be pain; secondary outcomes will be quality of life and adverse effects. Searches will be performed in Cochrane, Embase, LILACS, MEDLINE (via PubMed), and Scopus, plus gray literature (Google Scholar, ProQuest), reference lists, expert consultations, and ClinicalTrials.gov. Retrieved records will be merged in a reference manager, with duplicates removed, and imported into LASER AI software. Calibrated authors will independently select studies in two phases: title/abstract screening and full-text screening. Included studies will undergo narrative synthesis, and, if sufficiently homogeneous, meta-analysis. Risk of bias will be assessed with RoB 2, and certainty of evidence graded with GRADE.

## Introduction

Patient adherence to orthodontic treatment can be influenced by pain and changes in quality of life, making it essential to address patients’ concerns and provide adequate management [1–3]. Cooperation may decrease after appliance placement, as pressure and intermittent pain are common, especially with fixed appliances [1]. During the first three months of treatment, pain peaks between 24 h and 3 days, and analgesic use reflects moderate pain perception [2]. Appliance type also affects discomfort, with fixed and functional appliances causing more pain than simple removable plates [3]. Psychological factors strongly influence adaptation, and managing expectations helps maintain adherence [1–3].

Low-Level Laser Therapy (LLLT) is a complementary dental treatment that benefits hard and soft oral tissues with few side effects [4]. It delivers low-intensity photons that modulate cellular activity without generating heat, promoting tissue repair and analgesia [5,6]. The main challenge is defining optimal parameters to achieve consistent clinical outcomes [6].

Evidence suggests that LLLT can alleviate pain after orthodontic activation, but variability in protocols, study quality, and possible placebo effects limit certainty [7–10]. For example, irradiation at 980 nm, 2.5 W/cm² and 600 J has shown significant pain reduction [11]. Nevertheless, the overall efficacy of LLLT for orthodontic pain remains uncertain, and standardized protocols are lacking [12].

Orthodontic pain is a frequent adverse effect of fixed appliances, aligners, separators, and orthopedic forces, with peak intensity 24–48 h after activation [13,14]. Up to 95% of patients experience pain from the inflammatory response involving cytokines, prostaglandins, and neuropeptides [15–18]. Since this mechanism is well established, identifying effective interventions is critical to improve adherence and comfort [1–3]. LLLT is a promising, non-invasive option with minimal adverse effects, yet studies show conflicting results and practical aspects such as cost and treatment time require consideration [4–8,19–21].

A recent randomized clinical trial (RCT) comparing low-level laser therapy with paracetamol–caffeine demonstrated clinically meaningful pain reductions, highlighting the need for a comprehensive synthesis of the available evidence [22]. Although two recent systematic reviews [20,23] examined orthodontic pain control, important gaps remain. One of them pooled several physical interventions without isolating LLLT, did not stratify by appliance type or laser parameters, and did not present certainty of the evidence [20], while another one focused only on separator-related pain and provided low-to-moderate certainty of evidence [23]. Neither incorporated a prospectively registered, peer-reviewed search with broad database and grey literature coverage, justifying a more comprehensive and methodologically rigorous review [20,23]. This review will also encompass different orthodontic treatment phases (such as separator placement, fixed appliance activation, and clear aligner insertion) to ensure a comprehensive synthesis of pain control across clinical scenarios.

Therefore, a new systematic review is needed to address these gaps. This review will evaluate the efficacy of LLLT for pain relief and postoperative sensitivity in orthodontic patients.

## Methods

The protocol for this review was developed following the PRISMA-P (Preferred Reporting Items for Systematic Review and Meta-Analysis Protocols) guidelines [24]. It was registered in the International Prospective Register of Systematic Reviews (PROSPERO) under the number CRD420251033037. A systematic review of intervention studies will be conducted by the methodology proposed in the Cochrane Handbook [25].

### Study status and timeline

A preliminary search strategy was developed and pilot-tested to refine keywords, indexing terms, and database-specific adaptations. However, the full electronic search has not yet been conducted. The complete and final search is planned for June 2026, after which study selection, data extraction, and subsequent stages of the review will proceed according to the predefined protocol. The protocol is being submitted before the final search to ensure methodological transparency and avoid selective reporting, in accordance with PRISMA-P recommendations.

### Research question

“What is the efficacy of low-level laser therapy in alleviating pain, compared to other interventions, sham therapy, or no treatment, in patients undergoing orthodontic treatment?”

The research question was formulated based on the PICOS acronym (Population, Intervention, Comparison, Outcome, and Study Design), as outlined in Table 1.

**Table 1:** PICOS Framework for the Research Question.

Population	Orthodontic patients undergoing
**Intervention**	Low-Level Laser Therapy (LLLT)
**Comparison**	Other interventions, sham therapy or no treatment
**Outcome**	*Primary outcomes:* duration and intensity of pain *Secondary outcomes:* quality of life, adverse effects
**Study Design**	Randomized controlled trials

### Eligibility Criteria

#### Inclusion criteria.

Orthodontic patients of all ages will be included, whether undergoing treatment with fixed appliances or clear aligners. The intervention of interest is LLLT, encompassing various types and wavelengths, applied to the alveolar region. LLLT is considered within photobiomodulation therapy. However, this review will include only laser-based interventions and exclude LED-based PBMT due to differences in dosimetry and coherence. Eligible comparators will include other interventions, sham therapy, and no treatment. The primary outcome is pain intensity. Secondary outcomes include quality of life and patient satisfaction, and adverse effects of LLLT. Randomized controlled trials, including split-mouth and crossover designs (considering only data from the first assessment), will be included. No restrictions will be applied regarding language or publication date. If multiple tools are used to measure an outcome, the one most commonly adopted in the literature will be prioritized.

### Exclusion criteria

1. Studies involving specific treatments such as hybrid orthodontics or skeletal anchorage. These exclusions aim to reduce clinical heterogeneity, as skeletal anchorage and intermaxillary elastics involve different pain mechanisms that may confound the isolated effect of LLLT.
2. Studies involving patients under continuous use of medications (e.g., analgesics, anti-inflammatory drugs, corticosteroids) that could influence pain perception or confound the effect of the laser.
3. Studies in which the effect of the laser cannot be isolated from other interventions.
4. Reviews, letters, books, conference abstracts, case reports, opinion articles, technique articles, posters, and clinical practice guidelines;
5. Full text is unavailable, even after contacting the corresponding authors (three attempts in 3 weeks). Additional efforts will include contacting co-authors and searching repositories; if unavailable, data may be described narratively.

### Search Methods for Study Identification

#### Information sources and search strategy.

A preliminary search strategy for PubMed was developed by the first author (C.R.L.) and improved with the assistance of a librarian. This strategy was peer-reviewed using the PRESS (Peer Review of Electronic Search Strategies) guideline to ensure accuracy and comprehensiveness by a second librarian. The peer-reviewed PubMed search strategy, provided verbatim (APPENDIX 1), will serve as the basis for and be adapted to the other databases. The search will be applied to the Cochrane, Embase, LILACS (in Spanish: *Literatura Latinoamericana y del Caribe en Ciencias de la Salud*), MEDLINE (via PubMed), and Scopus databases. Additionally, searches will be conducted in gray literature via ProQuest, Google Scholar, and Clinical Trials. Experts will be consulted, and references from included articles will be manually checked.

### Study records

#### Data management.

The first author (C.R.L.) will compile all references into a single file and remove duplicates into a reference software manager (EndNote-Web^TM^, Clarivate^TM^, Jersey, USA).

### Methods for study selection

The selected studies will be imported into a screening platform, and the selection process will be conducted in two phases using LASER AI software (Evidence Prime Inc., https://www.evidenceprime.com/laser). In the first phase, three reviewers (C.R.L., D.B.O., and A.C.P.T.) will independently select titles and abstracts; in the second phase, they will apply the eligibility criteria to the full texts. A fourth reviewer (H.P.) will address discrepancies through consensus. The process will be sufficiently detailed to complete the PRISMA flow diagram [26].

### Data collection process

Data extraction using LASER AI will be conducted semi-automatically, integrating machine learning with human validation. Initially, the AI will be trained using initial manual annotations. Subsequently, the AI will process the articles and suggest extracted data. One reviewer (C.R.L.) will verify and adjust the extracted information, while two other reviewers (D.B.O. and A.C.P.T.) will reevaluate the corrections and identify any discrepancies. In cases of conflicts, a fourth reviewer (H.P.) will act as an arbitrator to facilitate consensus. Artificial intelligence (AI) tools (LASER AI, Evidence Prime Inc., and ChatGPT, OpenAI, model version Plus) will be used exclusively as assistive tools. All AI outputs will be independently verified by human reviewers. Prompts, outputs, and corrections will be documented to ensure auditability and reproducibility. AI will not be used for final decision-making. Once validated, the final data will be exported in Microsoft Excel spreadsheet format (Microsoft Corporation, Redmond, WA, USA) for statistical analysis (APPENDIX 2), thereby enhancing efficiency and standardization. If data are missing, the study authors will be contacted. Data from graphs can be extracted using WebPlotDigitizer (https://automeris.io/WebPlotDigitizer/) [27]. The data will be presented in tables in the article. Reports originating from the same study will be analyzed collectively.

### Data items

The variables to be extracted will be predefined, including study characteristics (author and year), study design, sample size (with sex distribution), and age (mean and standard deviation). Intervention groups will be categorized as laser, sham therapy, or control. Laser parameters such as wavelength (nm), power, irradiance, energy density, spot size, pulse mode, application time, number os sessions, irradiation sites, and dose (Joules) will also be extracted. Additionally, outcome measurement instruments, pain assessment time points, and results (mean and standard deviation) will be recorded. The results will specifically address the placement of separator elastics and the activation of orthodontic mechanics, with pain intensity evaluated at four predefined time points: T1 (up to 24 hours post-intervention), T2 (more than 24–48 hours), T3 (more than 48 hours to 7 days), and T4 (after 7 days). Meta-analyses will be conducted separately for each of these time points. If data for T3 or T4 are not reported, only T1 and T2 will be included.

### Outcomes and prioritization

The primary outcome is pain intensity, measured using the Visual Analog Scale (VAS) at the specified time points. Secondary outcomes include quality of life and patient satisfaction, assessed with the OHIP-14 (Oral Health Impact Profile) [26,27], analgesic consumption (when reported), and LLLT adverse effects. The latter will be recorded using a systematic checklist that categorizes events by type (e.g., headache, irritation) and intensity (mild, moderate, severe).

### Risk of bias analysis

The risk of bias will be independently assessed by two reviewers (C.R.L. and D.B.O.) using the Cochrane RoB 2 tool [28]. Given that the use of AI in evidence synthesis is evolving and requires caution, with evidence suggesting variability in accuracy compared to human reviewers (Taneri PE, 2025), AI such as ChatGPT (ChatGPT, OpenAI, 2024) [29] will be used only as an assistive tool. Specifically, ChatGPT will aid the reviewers by processing specific commands to answer questions for each domain of the tool. All AI outputs will be verified independently by three authors (C.R.L., D.B.O., and A.C.P.T.). Disagreements will be resolved through consensus meetings or adjudication by a fourth reviewer (H.P.). The evaluation will cover biases related to randomization, deviations from intended interventions, incomplete outcome data, outcome measurement, and reporting results, with the risk categorized as high, low, or “some concerns.” Judgments will be presented in tables using RevMan (RevMan 5 (Review Manager) Version 5.4 [Software] The Cochrane Collaboration, 2020) and included in the forest plot of the meta-analyses. Studies will be classified as having a low risk of bias if all key domains are rated as low risk, as having some concerns if any domain is rated as some concerns, and as high risk if any domain is rated as high risk.

### Data synthesis

A narrative synthesis of the included studies will be conducted, with an additional quantitative synthesis through meta-analyses if sufficient clinical and methodological homogeneity is identified. Meta-analyses will be performed using RevMan 5.4 (Review Manager, The Cochrane Collaboration, 2020), and a random-effects model will be applied. The specific effect measures (Relative Risks, Mean Differences, and Standartized Mean Differences) are detailed in the Effect Measures sub-section. Statistical heterogeneity will be assessed using the Chi² test (P < 0.10) and quantified with the I² statistic; between-study variance (τ²) will also be reported when available. For multi-arm trials, relevant intervention groups will be combined when appropriate, or comparator groups will be split to avoid double-counting. When multiple timepoints are reported, outcomes will be grouped according to predefined time windows, and a single timepoint per study will be selected for each window based on proximity to the defined interval.

If additional analyses beyond the capabilities of RevMan are required, statistical analyses may be supplemented using R software (e.g., meta or metafor packages).

### Effect measures

For dichotomous outcomes, results will be reported as RR with 95% confidence intervals (CI). For continuous outcomes, results will be expressed as mean differences (MD) when the same scale is used or as standardized mean differences (SMD) for different scales, with 95% CI. When scales differ, standardized mean differences will be used. VAS 0–100 mm scores will be converted to a 0–10 scale. The mean and standard deviation for each group will be calculated relative to the baseline and follow-up measurements using RevMan (RevMan 5 (Review Manager) Version 5.4 [Software] The Cochrane Collaboration, 2020). The SMD will be interpreted as a small effect (0.2), moderate effect (0.5), or large effect (0.8) [30].

### Unit of analysis

The unit of analysis will be the patient for randomized controlled trials and each side of the patient for split-mouth studies. The split-mouth studies will be grouped separately in different meta-analyses and will use paired analyses when possible. If the correlation is unavailable, it will be imputed and tested in sensitivity analyses. For crossover trials, only first-period data will be used.

### Handling of missing data

The corresponding author of the original studies will be contacted via email to obtain any missing data. If the data are not provided, they will be considered as missing. Only the available data will be analyzed. Missing SDs will be estimated from available statistics, and medians will be converted when appropriate. Sensitivity analyses will be performed to assess how sensitive the results will be to reasonable changes in the assumptions made [31]. All correspondence with the authors will be documented.

### Assessment of heterogeneity

Random-effects models will be used, with I² and τ² reported when available. Statistical heterogeneity will be assessed across the results of different trials using the Chi-squared (Chi²) test, with significance defined as P < 0.1. The I² statistics will quantify heterogeneity among the studies in each analysis. Heterogeneity will be categorized as “not important” (0% to 40%), moderate (30% to 60%), substantial (50% to 90%), and considerable (75% to 100%) [32]. Additionally, heterogeneity will be visually assessed by inspecting the overlap of CIs and may be explored through subgroup analyses.

### Subgroup analysis

To minimize multiplicity and focus on clinically relevant heterogeneity, subgroup analyses will be limited to three prespecified factors: [1] orthodontic appliance type (separator elastics, fixed appliance activation, removable appliance activation, or new aligner insertion); [2] laser type (low-level laser therapy vs. diode laser); and [3] wavelength (e.g., 660, 808, 940 nm). Additional subgroup analyses may include energy density, number of sessions, and comparator type if data allow. Analyses will be conducted as reported by the included studies.

Regarding comparator handling, each distinct active comparator will be assessed in its own meta-analysis. Different non-pharmacologic interventions will not be pooled in a single model, except for sham/placebo, which may be combined. Pharmacologic comparators may be pooled only if they belong to the same therapeutic class (e.g., different nonsteroidal anti-inflammatory drugs); otherwise, they will be analyzed separately.

### Sensitivity analysis

A sensitivity analysis will be performed to assess the robustness of the overall effect estimate. This analysis will examine the influence of studies with a high risk of bias and small sample sizes on the overall effect estimate. This will be done by removing studies with these characteristics from each meta-analysis to assess their influence on the results. Sensitivity analyses will include exclusion of high-risk studies, imputed data, and outliers.

### Assessment of publication bias

If more than 10 studies are included in any comparison, publication bias will be assessed through funnel plot analysis and using Egger’s test [33].

### Certainty of evidence assessment

The certainty of the evidence will be evaluated according to the Grading of Recommendations, Assessment, Development, and Evaluation (GRADE) approach [34]. The assessment process will be conducted by the first author (C.R.L.) and verified by the second and third authors (A.C.P.T. and D.B.O.). A summary of the findings table will be created using the GRADEpro GDT software (GRADEpro: Guideline Development Tool, 2015) [35]. Five domains for downgrading the certainty will be evaluated: risk of bias, inconsistency, indirect evidence, imprecision, and publication bias. These domains will be classified as “not serious,” “serious,” or “very serious.” The certainty of the evidence will be categorized as high, moderate, low, or very low.

### Expected results and impacts

Pain is a common concern among patients undergoing orthodontic treatment, affecting their quality of life and treatment adherence. The LLLT presents itself as a non-invasive approach to pain relief and healing improvement, offering a promising alternative without the side effects of conventional medications. This study has the potential to assist healthcare professionals in clinical decision-making, enhancing treatment protocols and outcomes. A systematic review can identify knowledge gaps, guide future research, and develop evidence-based guidelines, leading to more effective treatments and greater patient satisfaction.

This systematic review aims to examine the evidence on the efficacy of laser therapy in reducing pain caused by orthodontic treatment [4]. The study stands out for its robust methodology, including a comprehensive search of five databases, grey literature, reference lists, and collaboration with experts, ensuring the reliability of the results. The transparent methodology facilitates replication, allowing independent validation by other researchers [36]. The review employs three blinded reviewers (C.R.L., D.B.O., and A.C.P.T.), supported by a fourth (H.P.) to avoid selection bias. The analysis provides valuable insights for professionals and patients considering laser therapy, potentially influencing therapeutic decisions and improving informed consent. Despite potential limitations, such as the risk of bias, this review can serve as a valuable resource, contributing to a comprehensive understanding of the efficacy of laser therapy and guiding informed decisions on pain relief in orthodontic treatment.

### Strengths and limitations of this study

- This protocol is based on a comprehensive and peer-reviewed search strategy, including grey literature sources and expert consultation.
- It incorporates artificial intelligence tools (Laser AI and ChatGPT) combined with double human cross-checking to enhance efficiency and accuracy in study selection and data extraction.
- The variability in laser parameters and treatment protocols is expected to introduce heterogeneity, which may limit the feasibility of meta-analysis.
- Exclusion of studies without available full texts may introduce a risk of publication bias.

## Supporting information

S1 File PRISMA-P_completed. (DOCX)

## Abbreviations

PRESS: Peer Review of Electronic Search Strategies
LILACS: Latin American and Caribbean Health Sciences Literature
LLLT: Low-Level Laser Therapy
nm: nanometers
J: Joules
RCTs: Randomized Controlled Trials
VAS: Visual Analog Scale
ODs: Odds Ratio
RRs: Relative Risk
CI: Confidence Intervals
MD: Mean Differences
SMD: Standardized Mean Differences
GRADE: Grading of Recommendation, Assessment, Development, and Evaluation
