# Supplementary material for: Efficacy of low-level laser therapy in pain relief during orthodontic treatment: Protocol for a systematic review and meta-analysis
Source: PLoS One. 2026 Jul 9;21(7):e0352702. doi: 10.1371/journal.pone.0352702 (PMC13349292; doi:10.1371/journal.pone.0352702)
Supplement: Attachment — Submitted filename: Response to Reviewers.docx [file pone.0352702.s003.docx]

# **Response to Reviewers**

**Manuscript ID:** PONE-D-25-63924
**Title:** *Low-Level Laser Therapy for Orthodontic Pain Relief: Protocol for a Systematic Review and Meta-analysis*

Dear Editor and Reviewers,

We thank the Academic Editor and the reviewers for their careful evaluation and constructive comments. We have revised the manuscript accordingly and believe these changes have substantially improved its clarity, methodological rigor, and transparency.

All modifications have been incorporated into the revised manuscript and are highlighted in yellow in the tracked-changes version. Below, we provide a detailed, point-by-point response to each comment.

# **GENERAL COMMENTS (EDITORIAL REQUIREMENTS)**

**Comment:** Data availability, funding inconsistencies, supporting information, anonymization, and formatting issues.

**Response:**
We have addressed all editorial requirements:

- The Data Availability Statement was expanded to specify repository, content, and reproducibility elements.
- Funding information has been corrected and aligned across all sections.
- Supporting Information captions were added at the end of the manuscript.
- The PRISMA file was anonymized and resubmitted in compliance with PLOS data policy.
- The manuscript was revised to comply fully with PLOS formatting guidelines.

# **REVIEWER #1**

### **1. Statistical and methodological details missing**

**Comment:** Lack of specification regarding timepoints, effect measures, models, handling of split-mouth/crossover designs, heterogeneity, and subgroup analyses.

**Response:**
We agree and have now fully specified the statistical framework:

- Predefined time windows for pain outcomes (T1–T4) were introduced.
- Effect measures clarified (RR, MD, SMD) with harmonization rules.
- Random-effects model adopted with I² and τ² reporting.
- Handling of split-mouth and crossover designs is explicitly defined.
- Detailed plans for multi-arm trials and multiple timepoints included.
- Expanded subgroup and sensitivity analyses.

These changes are reflected in:

- *Methods → Data Items, Effect Measures, Unit of Analysis, Data Synthesis, Subgroup Analysis, Sensitivity Analysis*

### **2. AI transparency and reproducibility**

**Comment:** Insufficient detail on AI tools (LASER AI and ChatGPT).

**Response:**
We have substantially expanded the AI methodology:

- Defined AI as assistive only, with no decision-making role.
- Included human verification at all stages.
- Added documentation of prompts, outputs, and corrections.
- Included auditability and reproducibility procedures.

Additionally, we incorporated supporting literature regarding AI limitations.

Location:

- *Methods → Data Collection Process*
- *Methods → Risk of Bias Analysis*

### **3. Data extraction and missing data handling**

**Comment:** Lack of clarity on extracted variables and missing data procedures.

**Response:**
We have added:

- A comprehensive list of extracted variables, including full laser dosimetry parameters.
- Procedures for contacting authors.
- Rules for imputation (SDs, medians) and sensitivity testing.

Location:

- *Methods → Data Items*
- *Methods → Handling of Missing Data*

### **4. Justification of exclusion criteria**

**Comment:** Exclusion of skeletal anchorage and elastics insufficiently justified.

**Response:**
We clarified that:

- These interventions involve distinct pain mechanisms, which may confound the isolated effect of LLLT.
- The aim is to preserve clinical homogeneity.

Location:

- *Methods → Exclusion Criteria*

### **5. LED vs LLLT ambiguity**

**Comment:** Clarify inclusion of PBMT/LED.

**Response:**
We clarified that:

- LLLT is considered within PBMT, but
- Only laser-based interventions are included, excluding LED-based PBMT due to differences in coherence and dosimetry.

Location:

- *Methods → Eligibility Criteria*

### **6. Inclusion of analgesic consumption**

**Comment:** Clinically relevant outcome missing.

**Response:**
We have added analgesic consumption as a secondary outcome.

Location:

- *Methods → Outcomes and Prioritization*

# **REVIEWER #2**

### **1. Title revision**

**Comment:** Title should include meta-analysis.

**Response:**
Revised as:

*Low-Level Laser Therapy for Orthodontic Pain Relief: Protocol for a Systematic Review and Meta-analysis*

### **2. AI workflow transparency**

**Response:**
Addressed as described above (Reviewer #1, Point 2), including:

- Versioning
- Validation
- Reproducibility

### **3. Search timing (2026)**

**Comment:** Clarify why protocol precedes search.

**Response:**
We added explicit justification:

- Submission prior to search ensures transparency and avoids selective reporting, per PRISMA-P.

Location:

- *Methods → Study Status and Timeline*

### **4. Search strategy completeness**

**Response:**
We confirmed that:

- Full PubMed strategy is included verbatim in Appendix 1
- Adaptations for other databases will be provided

### **5. Exclusion of full-text unavailable studies**

**Response:**
We expanded the process:

- Contact corresponding authors (3 attempts)
- Contact co-authors
- Search repositories
- If still unavailable → narrative inclusion when possible

### **6. Heterogeneity and subgroup analysis**

**Response:**
We expanded subgroup analyses to include:

- Wavelength
- Energy density
- Number of sessions
- Comparator type

### **7. Data availability**

**Response:**
We replaced the generic statement with:

- Explicit OSF repository deposition
- Inclusion of datasets, AI outputs, and code

### **8. Funding inconsistencies**

**Response:**
All funding information has been corrected and standardized across sections.

# **REVIEWER #3**

### **1. Data sharing details**

**Response:**
Now explicitly includes:

- Repository (OSF)
- Types of shared data (datasets, extraction forms, AI annotations, code)

### **2. Validation of AI tools**

**Response:**
We added:

- Explicit caution regarding AI reliability
- Citation of Taneri et al. (2025) on RoB assessment variability
- Reinforcement of mandatory human validation

### **3. Exclusion criteria and crossover rationale**

**Response:**
Clarified:

- Exclusion based on confounding pain mechanisms
- Use of first-period data only in crossover trials to avoid carryover effects

### **4. Minor issues (references, formatting, clarity)**

**Response:**
We conducted a complete revision of references, numbering, grammar, and formatting.

# **ADDITIONAL INSERTIONS REQUESTED BY REVIEWERS**

We incorporated all suggested relevant studies, including:

- Owayda et al. (RCT on LLLT vs paracetamol–caffeine)
- Al-Hanbali et al. (separator pain)

**These were inserted in the Introduction as appropriate.**

# **FINAL STATEMENT**

We believe that the revised manuscript now meets PLOS ONE methodological and reporting standards, and provides transparent, reproducible, and rigorous methodology, and also addresses all reviewer concerns in full.

We appreciate the reviewers’ insightful feedback and hope the revised manuscript is now suitable for publication.

Sincerely,

Patrícia Pauletto, on behalf of all authors

Contact: + 593 99900 6460
