# Supplementary material for: Efficacy of low-level laser therapy in pain relief during orthodontic treatment: Protocol for a systematic review and meta-analysis
Source: PLoS One. 2026 Jul 9;21(7):e0352702. doi: 10.1371/journal.pone.0352702 (PMC13349292; doi:10.1371/journal.pone.0352702)
Supplement: Peer review history

# Peer review: Decision Letter 0

**DOI:** 10.1371/journal.pone.0352702.r001

**Authors:** Spyridon Papageorgiou

11 Mar 2026

PONE-D-25-63924

Efficacy of Low-Level Laser Therapy in Pain Relief During Orthodontic Treatment: Protocol for a Systematic Review

PLOS One

Dear Dr. Pauletto,

Thank you for submitting your manuscript to PLOS ONE. After careful consideration, we feel that it has merit but does not fully meet PLOS ONE’s publication criteria as it currently stands. Therefore, we invite you to submit a revised version of the manuscript that addresses the points raised during the review process.

The reviewers have come in the meanwhile back with their comments on your submission. Based on this, I ask you to revise your manuscript and respond to all comments accordingly.

Please submit your revised manuscript by Apr 25 2026 11:59PM. If you will need more time than this to complete your revisions, please reply to this message or contact the journal office at plosone@plos.org. When you're ready to submit your revision, log on to https://www.editorialmanager.com/pone/ and select the 'Submissions Needing Revision' folder to locate your manuscript file.

Please include the following items when submitting your revised manuscript:

- A letter that responds to each point raised by the academic editor and reviewer(s). You should upload this letter as a separate file labeled 'Response to Reviewers'.
- A marked-up copy of your manuscript that highlights changes made to the original version. You should upload this as a separate file labeled 'Revised Manuscript with Track Changes'.
- An unmarked version of your revised paper without tracked changes. You should upload this as a separate file labeled 'Manuscript'.

If you would like to make changes to your financial disclosure, please include your updated statement in your cover letter. Guidelines for resubmitting your figure files are available below the reviewer comments at the end of this letter.

If applicable, we recommend that you deposit your laboratory protocols in protocols.io to enhance the reproducibility of your results. Protocols.io assigns your protocol its own identifier (DOI) so that it can be cited independently in the future. For instructions see: https://journals.plos.org/plosone/s/submission-guidelines#loc-laboratory-protocols. Additionally, PLOS ONE offers an option for publishing peer-reviewed Lab Protocol articles, which describe protocols hosted on protocols.io. Read more information on sharing protocols at https://plos.org/protocols?utm_medium=editorial-email&utm_source=authorletters&utm_campaign=protocols.

We look forward to receiving your revised manuscript.

Kind regards,

Spyridon N. Papageorgiou, DDS, Dr Med Dent

Academic Editor

PLOS One

Journal Requirements:

When submitting your revision, we need you to address these additional requirements.

1. Please ensure that your manuscript meets PLOS ONE's style requirements, including those for file naming. The PLOS ONE style templates can be found at

https://journals.plos.org/plosone/s/file?id=wjVg/PLOSOne_formatting_sample_main_body.pdf and https://journals.plos.org/plosone/s/file?id=ba62/PLOSOne_formatting_sample_title_authors_affiliations.pdf

2. Please provide a complete Data Availability Statement in the submission form, ensuring you include all necessary access information or a reason for why you are unable to make your data freely accessible. If your research concerns only data provided within your submission, please write "All data are in the manuscript and/or supporting information files" as your Data Availability Statement.

3. We note that the grant information you provided in the ‘Funding Information’ and ‘Financial Disclosure’ sections do not match.

When you resubmit, please ensure that you provide the correct grant numbers for the awards you received for your study in the ‘Funding Information’ section.

4. Please include captions for your Supporting Information files at the end of your manuscript, and update any in-text citations to match accordingly. Please see our Supporting Information guidelines for more information: http://journals.plos.org/plosone/s/supporting-information.

5. We note that there is identifying data in the Supporting Information file <PRISMA-P_completed.docx>. Due to the inclusion of these potentially identifying data, we have removed this file from your file inventory. Prior to sharing human research participant data, authors should consult with an ethics committee to ensure data are shared in accordance with participant consent and all applicable local laws.

Data sharing should never compromise participant privacy. It is therefore not appropriate to publicly share personally identifiable data on human research participants. The following are examples of data that should not be shared:

-Name, initials, physical address

-Ages more specific than whole numbers

-Internet protocol (IP) address

-Specific dates (birth dates, death dates, examination dates, etc.)

-Contact information such as phone number or email address

-Location data

-ID numbers that seem specific (long numbers, include initials, titled “Hospital ID”) rather than random (small numbers in numerical order)

Data that are not directly identifying may also be inappropriate to share, as in combination they can become identifying. For example, data collected from a small group of participants, vulnerable populations, or private groups should not be shared if they involve indirect identifiers (such as sex, ethnicity, location, etc.) that may risk the identification of study participants.

Additional guidance on preparing raw data for publication can be found in our Data Policy (https://journals.plos.org/plosone/s/data-availability#loc-human-research-participant-data-and-other-sensitive-data) and in the following article: http://www.bmj.com/content/340/bmj.c181.long.

Please remove or anonymize all personal information (<specific identifying information in file to be removed>), ensure that the data shared are in accordance with participant consent, and re-upload a fully anonymized data set. Please note that spreadsheet columns with personal information must be removed and not hidden as all hidden columns will appear in the published file.

6. If the reviewer comments include a recommendation to cite specific previously published works, please review and evaluate these publications to determine whether they are relevant and should be cited. There is no requirement to cite these works unless the editor has indicated otherwise.

[Note: HTML markup is below. Please do not edit.]

Reviewers' comments:

Reviewer's Responses to Questions

**Comments to the Author**

1. Does the manuscript provide a valid rationale for the proposed study, with clearly identified and justified research questions?

The research question outlined is expected to address a valid academic problem or topic and contribute to the base of knowledge in the field.

Reviewer #1: Yes

Reviewer #2: Yes

Reviewer #3: Yes

**********

2. Is the protocol technically sound and planned in a manner that will lead to a meaningful outcome and allow testing the stated hypotheses?

The manuscript should describe the methods in sufficient detail to prevent undisclosed flexibility in the experimental procedure or analysis pipeline, including sufficient outcome-neutral conditions (e.g. necessary controls, absence of floor or ceiling effects) to test the proposed hypotheses and a statistical power analysis where applicable. As there may be aspects of the methodology and analysis which can only be refined once the work is undertaken, authors should outline potential assumptions and explicitly describe what aspects of the proposed analyses, if any, are exploratory.

Reviewer #1: Yes

Reviewer #2: Partly

Reviewer #3: Yes

**********

3. Is the methodology feasible and described in sufficient detail to allow the work to be replicable?

Descriptions of methods and materials in the protocol should be reported in sufficient detail for another researcher to reproduce all experiments and analyses. The protocol should describe the appropriate controls, sample size calculations, and replication needed to ensure that the data are robust and reproducible.

Reviewer #1: Yes

Reviewer #2: No

Reviewer #3: Yes

**********

4. Have the authors described where all data underlying the findings will be made available when the study is complete?

The PLOS Data policy requires authors to make all data underlying the findings described in their manuscript fully available without restriction, with rare exception, at the time of publication. The data should be provided as part of the manuscript or its supporting information, or deposited to a public repository. For example, in addition to summary statistics, the data points behind means, medians and variance measures should be available. If there are restrictions on publicly sharing data—e.g. participant privacy or use of data from a third party—those must be specified.

Reviewer #1: Yes

Reviewer #2: Yes

Reviewer #3: No

**********

5. Is the manuscript presented in an intelligible fashion and written in standard English?

PLOS ONE does not copyedit accepted manuscripts, so the language in submitted articles must be clear, correct, and unambiguous. Any typographical or grammatical errors should be corrected at revision, so please note any specific errors here.

Reviewer #1: Yes

Reviewer #2: No

Reviewer #3: Yes

**********

6. Review Comments to the Author

Please use the space provided to explain your answers to the questions above and, if applicable, provide comments about issues authors must address before this protocol can be accepted for publication. You may also include additional comments for the author, including concerns about research or publication ethics.

You may also provide optional suggestions and comments to authors that they might find helpful in planning their study.

(Please upload your review as an attachment if it exceeds 20,000 characters)

Reviewer #1: This protocol outlines a planned systematic review and (conditional) meta-analysis to assess the efficacy of low-level laser therapy (LLLT; photobiomodulation) in reducing orthodontic pain across different treatment scenarios (e.g., separators, fixed appliance activation, aligner insertion). The authors will include randomized controlled trials, compare LLLT against sham/no treatment/other interventions, and evaluate primary (pain intensity/duration) and secondary outcomes (quality of life, adverse effects), using RoB 2 and GRADE to appraise risk of bias and certainty of evidence.

Technical novelty and innovation

The protocol focuses specifically on LLLT for orthodontic pain (rather than pooling heterogeneous physical modalities), aiming to stratify by orthodontic phase and laser parameters—an important refinement over prior reviews.

Explicit plan to use GRADE and RoB 2 should enhance methodological transparency and utility for clinicians.

Inclusion of gray literature and expert consultation increases comprehensiveness and mitigates publication bias.

Experimental rigor and validation

PROSPERO registration and adherence to PRISMA-P indicate prospective methodological planning and reporting.

Two-phase independent screening with calibration and duplicate processes is appropriate for minimizing selection bias.

Clarity of presentation

The clinical problem and rationale for a focused, up-to-date synthesis are well motivated; the target population, interventions, comparators, and outcomes are clearly defined at a high level.

Significance of contributions

Orthodontic pain is a common, clinically significant issue; clarifying the effectiveness and parameters of LLLT has direct implications for patient comfort, adherence, and clinical decision-making.

Technical limitations or concerns

Important statistical/analytical details are missing: prespecified timepoints for pain outcomes; choice of effect size metrics; handling of split-mouth/crossover designs; statistical model (random- vs fixed-effects); subgroup and sensitivity analyses; small-study effects assessment.

Exclusion criteria omit justification for excluding skeletal anchorage and intermaxillary elastics broadly despite their prevalence and clinical relevance; this may limit applicability.

Ambiguity about whether LED-based PBMT is included/excluded (modern PBMT often uses LEDs in addition to lasers).

Experimental gaps or methodological issues

The planned use of AI tools (LASER AI and ChatGPT) is not sufficiently detailed (versioning, tasks, safeguards, human oversight, reproducibility), raising transparency and bias concerns.

No explicit plan for data extraction items (laser parameters, dosimetry details, analgesic consumption), missing data handling, or contacting authors for outcome data.

No clear plan for dealing with multiple arms/timepoints (e.g., several wavelengths/doses or repeated measurements) and to prevent unit-of-analysis errors.

Clarity or presentation issues

Minor inconsistencies/typos (e.g., software name “LASER Al,” parameter notation “2.5 W/cm^2 and 600 J”), and PROSPERO ID formatting should be corrected; some sections are truncated (e.g., search strategy details).

Technical soundness evaluation

The PICOS framework is appropriate, but the protocol lacks essential pre-specifications:

Outcomes: Define primary timepoints (e.g., 6h, 24h, 48h, 72h, 7 days) and whether a composite (e.g., area under the pain-time curve) will be used. Predefine acceptable pain scales and how to convert heterogeneous scales; consider minimally important difference thresholds where available.

Effect measures: State the primary effect measure (MD for homogeneous scales like 0–100 mm VAS; SMD if mixed scales). Specify handling of skewed data, medians/IQR, and imputation of SD.

Design issues: For split-mouth trials, specify paired analyses and the approach to within-person correlation (use reported correlations, calculate from paired data, or impute with sensitivity analyses). For crossover trials, confirm use of first-period data only and explain how carryover will be managed.

Multiple arms/timepoints: Predefine how to select among multiple laser doses/wavelengths or multiple comparator arms (avoid double counting by appropriate arm combination or comparator splitting). For multiple post-intervention timepoints, either prespecify primary timepoint(s) or meta-analyze time windows separately.

Heterogeneity and meta-analysis model: State that a random-effects model (e.g., REML with Hartung-Knapp adjustment) will be used given expected clinical/methodological heterogeneity; report I² and τ²; plan meta-regression only if k is sufficient and covariates are prespecified (e.g., wavelength, irradiance, energy density, pulsing, number of sessions).

Subgroups/sensitivity analyses: Prespecify subgroups by treatment phase (separators vs first archwire activation vs aligners), wavelength (e.g., red vs NIR), energy density, irradiance, pulsing vs continuous-wave, application site (buccal vs buccal+palatal), number of sessions, comparator type (sham vs usual care), age group (adolescents vs adults), and risk of bias strata. Sensitivity analyses: exclude high RoB trials, imputed correlations/SDs, non-peer-reviewed sources, and outliers/influential studies.

Small-study effects/publication bias: Plan funnel plots and Egger’s regression (or Peters/Macaskill) where ≥10 studies per analysis; consider contour-enhanced plots.

Eligibility criteria:

Clarify and justify exclusions of skeletal anchorage, hybrid orthodontics, and intermaxillary elastics; these are common and clinically relevant. If the aim is to maintain homogeneity, consider including them but analyzing in prespecified subgroups—or justify exclusion with respect to differential pain mechanisms.

Clarify whether LED-based PBMT will be included (contemporary PBMT includes lasers and LEDs). Align terminology: LLLT vs PBMT.

Experimental evaluation assessment

Study selection and data extraction: Provide full details on calibration (pilot rounds and target kappa), number of reviewers for extraction, conflict resolution, and standardized extraction forms. Explicitly list data items: participant characteristics, appliance type, orthodontic procedure, comparator(s), pain measurement timing/instruments, analgesic use, LLLT parameters (device type, wavelength, power, power density, spot size/beam area, energy, energy density, pulse parameters/duty cycle, application points, time per point, sessions, contact mode), and adverse effects definitions/collection method.

Missing/incomplete data: State procedures for contacting authors (attempts, time windows) not only for full texts but also for missing outcome or parameter data; describe imputation rules where applicable.

Certainty of evidence: Identify which outcomes/timepoints will populate Summary of Findings tables; specify GRADE domains and how imprecision thresholds will be judged (e.g., optimal information size).

Comparison with related work (using the summaries provided)

Prior reviews have combined heterogeneous physical modalities or limited scope (e.g., separator pain only) and often did not grade certainty. Your plan to focus on LLLT across treatment phases and apply GRADE is appropriate. Additionally, contemporary PBMT trials in orthodontics and related dental applications exist (e.g., mini-implant peri-implant soft tissue outcomes), highlighting parameter sensitivity and the need for careful dosimetry capture—your planned parameter-level extraction/subgrouping will be valuable if explicitly prespecified.

Mechanistic reviews of LLLT/PBMT emphasize dose-response and wavelength dependence; bringing these into prespecified subgroup analyses may yield clinically actionable insights (e.g., red vs NIR wavelengths, pulsed vs continuous delivery).

Discussion of broader impact and significance

If executed with the suggested methodological refinements, this review can meaningfully guide clinical protocols (dosimetry, timing, and application points) and patient counseling about expected pain trajectories and adverse effects. The inclusion of quality-of-life and adverse event outcomes enhances translational value. Clear reporting of laser parameters will also support harmonization of future trials.

Questions for Authors

Will LED-based photobiomodulation trials (PBMT) be included, or are you restricting the review strictly to laser devices? Please clarify terminology and inclusion criteria (LLLT vs PBMT).

What are the prespecified primary timepoints for pain assessment (e.g., 24h, 48h, 72h)? Will you analyze time windows separately or use area-under-the-curve metrics?

Which effect size metrics will you use (MD vs SMD), and how will you handle heterogeneous pain scales, medians/IQR, and missing SDs?

How will you analyze split-mouth RCTs (paired analyses and correlation coefficients) and manage crossover trials (beyond first-period-only inclusion)? Please specify correlation imputation and planned sensitivity analyses.

What is your planned meta-analytic model (random-effects vs fixed) and heterogeneity metrics (I², τ²)? Will you use Hartung-Knapp adjustments?

Which subgroup and sensitivity analyses will be prespecified (e.g., wavelength, energy density, irradiance, pulsing, number of sessions, orthodontic procedure, comparator, age, RoB strata)?

How will you manage multi-arm trials (multiple doses/wavelengths) and multiple timepoints to avoid double counting and multiplicity?

Will analgesic consumption be included as a secondary outcome, given its frequent reporting and clinical relevance?

Please provide more detail on the AI tools: versions, specific tasks (screening, extraction), thresholds for human override, validation steps, data privacy safeguards, and how you will ensure reproducibility and auditability of AI-assisted decisions.

The protocol excludes studies involving skeletal anchorage and intermaxillary elastics. Could you justify these exclusions, or consider including them with planned subgroup analyses?

Reviewer #2: Manuscript title (revise to reflect content):

===========

Low‑Level Laser Therapy for Orthodontic Pain Relief: Protocol for a Systematic Review and Meta‑analysis

Small summary

==========

This manuscript describes a PRISMA‑P–based protocol (PROSPERO CRD420251033037) to systematically review randomized trials assessing low‑level laser therapy (LLLT) for pain control during orthodontic treatment. The authors plan broad database and grey‑literature searches, AI‑assisted screening/extraction (LASER AI and ChatGPT), RoB 2 risk‑of‑bias assessment, and GRADE certainty appraisal. The protocol outlines eligibility, outcomes (pain, QoL, adverse effects), and planned meta‑analyses by predefined time points.

Major points

========

(1) Title clarity: The current short title understates the planned meta‑analytic intent; change to include “systematic review and meta‑analysis” for accuracy.

(2) AI methods transparency: The protocol relies heavily on LASER AI and ChatGPT for screening/extraction but lacks sufficient detail on training, validation, reproducibility, and how AI decisions will be audited and reported. Provide explicit performance metrics and a reproducible workflow.

(3) Search timing and completeness: The full electronic search is deferred to January 2026; the protocol should clarify why submission precedes the planned search and ensure the peer‑reviewed search strategy (Appendix) is complete and reproducible.

(4) eligibility/exclusion bias risk: Excluding studies with unavailable full texts after only three contact attempts risks bias; justify this threshold and describe alternative approaches (e.g., contacting co‑authors, using interlibrary loan, or including abstracts with caution).

(5) Heterogeneity handling: Laser parameter variability is acknowledged but the statistical plan lacks detail on subgroup/meta‑regression strategies (wavelength, dose, application timing, appliance type). Predefine subgroup analyses and thresholds for pooling.

(6) Outcome measurement harmonization: Prioritize and justify the pain scales and time points; specify methods for converting different scales (e.g., VAS to standardized mean difference) and handling crossover/split‑mouth designs.

(7) Funding and competing interests: Financial disclosure fields appear incomplete and inconsistent with the competing‑interests statement; fully disclose all funding sources, grant numbers, and any role of funders.

(8) Data availability and ethics: The Data Availability statement is generic; provide a concrete plan for sharing extracted datasets, AI training annotations, and analysis code to ensure reproducibility.

Minor points

========

(1) Title punctuation and wording: Remove redundancy in the running title and ensure it matches the main title.

(2) Appendices: Ensure the full PubMed search string (PRESS‑reviewed) is included verbatim in Appendix 1 and that database‑specific adaptations are shown.

(3) LASER AI vendor details: Provide version number, date of access, and any custom settings used.

(4) Risk of bias plan: Clarify how RoB 2 judgments will be reconciled (e.g., consensus meeting, third reviewer) and how domain‑level judgments will inform sensitivity analyses.

(5) Statistical software: State the software and packages to be used for meta‑analysis (e.g., RevMan, R metafor) and thresholds for heterogeneity (I² cutoffs).

(6) Grammar/formatting: Minor typographical errors and inconsistent numbering in the Methods should be corrected for readability.

Recommended insertions of closely related work

==============================

(1) Owayda et al. — A randomized controlled trial on the effectiveness of low‑level laser therapy versus paracetamol‑caffeine for pain control during overall orthodontic treatment (Sci Rep. 2025; doi: 10.1038/s41598-025-16658-2). Place: Introduction, paragraph summarizing existing RCT evidence for LLLT. Insertion sentence: “Notably, a recent randomized trial comparing LLLT with paracetamol‑caffeine reported clinically relevant pain reductions, underscoring the need for a comprehensive synthesis of randomized evidence (Owayda et al., 2025; doi: 10.1038/s41598‑025‑16658‑2).”

(2) Al‑Hanbali et al. — The effectiveness of low‑level laser therapy and low‑intensity pulsed ultrasound in reducing pain induced by orthodontic separation: a randomized controlled trial (BMC Oral Health. 2024; doi: 10.1186/s12903-024-03926-2). Place: Introduction, when discussing separator‑related pain and prior focused reviews. Insertion sentence: “Randomized evidence addressing separator‑related pain has been produced (Al‑Hanbali et al., 2024; doi: 10.1186/s12903‑024‑03926‑2), but prior syntheses have not consistently stratified by appliance or laser parameters.”

(3) Mousa et al. — Evaluation of low‑level laser therapy and piezocision in the en‑masse retraction of upper anterior teeth (Eur J Orthod. 2025; doi: 10.1093/ejo/cjaf026). Place: Methods or Discussion, when justifying exclusion criteria and the need to isolate laser effects from adjunctive surgical interventions. Insertion sentence: “Studies combining LLLT with surgical acceleration techniques (e.g., piezocision) highlight the difficulty of isolating laser effects, supporting our exclusion of multimodal interventions where laser impact cannot be separated (Mousa et al., 2025; doi: 10.1093/ejo/cjaf026).”

(4) Alam et al. — The Effect of Low‑Level Laser Therapy on Accelerating Tooth Movement in Orthodontic Patients: A Randomized Controlled Trial (J Pharm Bioallied Sci. 2024; doi: 10.4103/jpbs.jpbs_1168_24). Place: Discussion, when addressing secondary outcomes and biological plausibility (LLLT effects on tissue/biologic response). Insertion sentence: “LLLT’s biological effects on tooth movement and tissue response have been reported in randomized trials, which informs interpretation of pain outcomes alongside potential mechanistic effects (Alam et al., 2024; doi: 10.4103/jpbs.jpbs_1168_24).”

Reviewer #3: Overall, this is a rigorous protocol for a systematic review to determine the effectiveness of low-level laser therapy in alleviating pain during orthodontic treatment. The authors provide a good rationale for conducting the study. The proposed analyses are appropriate to address the research question. The writing is clear and concise.

Major

1) The authors indicate that they will share all data generated by the study; however, there is no information about where the data will be stored and shared.

2) Although the authors indicate that they will cross check information generated by artificial intelligence tools, I recommend providing additional information and references providing evidence of the validity of using a) Laser AI to extract data from included studies and b) ChatGPT to collect data for the risk of bias assessment. These are emerging technologies without a proven track record of accurately conducting such tasks. For example, the following study examined the validity of ChatGPT’s risk of bias assessment: Taneri PE. Human versus artificial intelligence: comparing Cochrane authors' and ChatGPT's risk of bias assessments. Cochrane Evidence Synthesis and Methods. 2025 Sep;3(5):e70044.

3) The authors could include additional information about the following:

a) Why are studies with specific treatments such as hybrid orthodontics with skeletal anchorage or intermaxillary elastics being excluded?

b) Rationale for only including the first assessment from studies using cross-over or split mouth designs

Minor

4) The authors should review reference numbers to ensure that they match the study referenced. For example, on line 90, there is mention of two recent systematic reviews (20,22). It looks like 22 is not a systematic review; however, 21 is a systematic review. Also, line 209, it is unclear how references 25 and 26 relate to the information in the sentence referenced; line 214, reference 27 relates to GRADE not the Cochrane RoB 2 tool mentioned in the sentence.

5) On line, 171, the authors reference a reading platform. Consider naming the platform that will be used.

**********

7. PLOS authors have the option to publish the peer review history of their article (what does this mean?). If published, this will include your full peer review and any attached files.

If you choose “no”, your identity will remain anonymous but your review may still be made public.

**Do you want your identity to be public for this peer review?**  For information about this choice, including consent withdrawal, please see our Privacy Policy.

Reviewer #1: **Yes:**  Ricardo Ney Cobucci

Reviewer #2: No

Reviewer #3: No

**********

[NOTE: If reviewer comments were submitted as an attachment file, they will be attached to this email and accessible via the submission site. Please log into your account, locate the manuscript record, and check for the action link "View Attachments". If this link does not appear, there are no attachment files.]

To ensure your figures meet our technical requirements, please review our figure guidelines: https://journals.plos.org/plosone/s/figures

You may also use PLOS’s free figure tool, NAAS, to help you prepare publication quality figures: https://journals.plos.org/plosone/s/figures#loc-tools-for-figure-preparation.

NAAS will assess whether your figures meet our technical requirements by comparing each figure against our figure specifications.

# Author comment: Author response to Decision Letter 1

**DOI:** 10.1371/journal.pone.0352702.r002

9 Jun 2026

Response to Reviewers

Manuscript Title: Adverse effects of removable orthodontic aligners: a systematic review with single-arm meta-analysis

Manuscript ID: PONE-D-26-03043

Dear Reviewers,

We thank you for the careful evaluation of our manuscript and for the constructive comments provided. We have revised the manuscript thoroughly to address all concerns. Below, we provide a detailed, point-by-point response indicating how each comment was addressed and where the corresponding revisions can be found in the manuscript.

All changes are reflected in the revised manuscript and highlighted in the tracked-changes version.

GENERAL CHANGES

The manuscript title was revised to explicitly reflect the single-arm meta-analysis approach, improving methodological transparency.

The Data Availability Statement was updated to include a public repository with DOI, ensuring full reproducibility.

Funding information was standardized and aligned with journal requirements.

Supplementary materials were expanded and cross-referenced.

The manuscript underwent full linguistic and editorial revision to ensure clarity and standard academic English.

REVIEWER #1

Comment 1 – Abstract

Please add details in background regarding the type of review conducted.

Response:

We revised the Abstract to explicitly state that this is a systematic review with single-arm meta-analysis.

Location: Abstract, Background and Methods sections.

Comment 2 – Introduction

Provide more details about adverse effects.

Response:

We expanded the Introduction to include a clearer and more comprehensive description of adverse effects (e.g., periodontal changes, enamel demineralization, speech alterations, TMD symptoms).

Location: Introduction, paragraphs describing adverse outcomes.

Comment 3 – Rationale for meta-analysis

Add rationale for the meta-analysis.

Response:

We explicitly justified the use of single-arm meta-analysis due to absence of consistent comparator groups and heterogeneity in reporting.

Location: End of Introduction and Synthesis Methods section.

Comment 4 – PRISMA flowchart

Add study flow-chart.

Response:

A PRISMA 2020 flow diagram was included and referenced in the Results section.

Location: Results – Study selection (Fig 2).

Comment 5 – Grey literature

Why include grey literature?

Response:

We clarified that grey literature was included to reduce publication bias, particularly relevant in adverse effects research.

Location: Information Sources section.

Comment 6 – Reviewer calibration

Add calibration details.

Response:

We described the calibration exercise conducted prior to screening and risk-of-bias assessment.

Location: Study Selection and Risk of Bias sections.

Comment 7 – Discussion (clinical implications)

Expand discussion on dehiscence/fenestration.

Response:

We expanded this section to include clinical implications and the importance of treatment planning and monitoring.

Comment 8 – Halitosis (long-term evidence)

Add literature beyond 4 months.

Response:

We incorporated additional discussion on longer-term findings and clarified the transient nature and limitations of current evidence.

REVIEWER #2

Major Comments

1. Title accuracy

Response:

Title revised to explicitly include single-arm meta-analysis.

Location: Title page.

2. Search date and eligibility clarity

Response:

We clarified:

Search date (May 4, 2025)

Inclusion of early online/in-press studies

Location: Methods – Information Sources and Study Selection.

3. Data availability and transparency

Response:

A complete and consistent Data Availability Statement was added, including:

OSF repository

DOI

Full datasets and R scripts

Location: Results – Data Availability Statement.

4. Funding and role of funders

Response:

Funding details were standardized and clarified.

A statement on funder role was added (no involvement in study design or analysis).

5. Use of AI tools

Response:

We clarified:

AI was used only to identify text excerpts

All decisions were made by human reviewers

Prompts were included in Supplementary Material

Location: Risk of Bias section + S4 File.

6. Heterogeneity and pooling justification

Response:

We strengthened the justification for pooling under high heterogeneity, explicitly referencing:

Conceptual consistency across studies

Use of random-effects models

Sensitivity analyses

Location: Synthesis Methods.

7. GRADE transparency

Response:

We added explicit justification for each GRADE rating and provided detailed evidence profiles.

8. Outcome harmonization

Response:

We expanded the harmonization description and added:

Stepwise procedures

Worked examples in Supplementary Material (S5)

Location: Synthesis Methods + S5.

9. Selective reporting

Response:

We conducted a structured assessment of selective reporting and included:

Dedicated supplementary file (S6)

Summary paragraph in Methods

Location: Synthesis Methods.

10. Clinical interpretation

Response:

We revised conclusions to avoid unsupported comparisons with fixed appliances, clarifying that:

Only aligner arms were analyzed

Direct comparisons require dedicated trials

Minor Comments

All minor comments were addressed:

Abstract updated with study design breakdown

PRISMA details clarified

Tables and figures standardized

Terminology harmonized (e.g., ARR vs root resorption)

Statistical details expanded (r = 0.5; sensitivity r = 0.3–0.7)

Imputed data handled and sensitivity analysis described

PROSPERO included in abstract

Language edited for clarity

Supplementary materials completed and cross-referenced

EDITORIAL REQUIREMENTS (PLOS ONE)

We confirm that all journal requirements were addressed:

Data and code fully available (OSF repository)

Funding statement corrected and standardized

Ethics statement located in Methods only

Supporting Information labeled and cited appropriately

Tables and figures included and properly formatted

FINAL STATEMENT

We believe the revised manuscript now fully addresses the reviewers’ concerns and aligns with PLOS ONE methodological and reporting standards. The revisions substantially improve transparency, methodological clarity, and reproducibility.

We appreciate the reviewers’ insightful feedback and remain available for any further clarification.

Sincerely,

Patrícia Pauletto on behalf of all authors

Contact: + 593 99900 6460

Attachment Submitted filename: Response to Reviewers.docx

# Editor report: Decision Letter 1

**DOI:** 10.1371/journal.pone.0352702.r003

**Authors:** Spyridon Papageorgiou, Spyridon Papageorgiou

14 Jun 2026

Efficacy of Low-Level Laser Therapy in Pain Relief During Orthodontic Treatment: Protocol for a Systematic Review and Meta-analysis

PONE-D-25-63924R1

Dear Dr. Pauletto,

We’re pleased to inform you that your manuscript has been judged scientifically suitable for publication and will be formally accepted for publication once it meets all outstanding technical requirements.

Within one week, you’ll receive an e-mail detailing the required amendments. When these have been addressed, you’ll receive a formal acceptance letter and your manuscript will be scheduled for publication.

An invoice will be generated when your article is formally accepted. Please note, if your institution has a publishing partnership with PLOS and your article meets the relevant criteria, all or part of your publication costs will be covered. Please make sure your user information is up-to-date by logging into Editorial Manager at Editorial Manager® and clicking the ‘Update My Information' link at the top of the page. For questions related to billing, please contact billing support.

If your institution or institutions have a press office, please notify them about your upcoming paper to help maximize its impact. If they’ll be preparing press materials, please inform our press team as soon as possible -- no later than 48 hours after receiving the formal acceptance. Your manuscript will remain under strict press embargo until 2 pm Eastern Time on the date of publication. For more information, please contact onepress@plos.org.

Kind regards,

Spyridon N. Papageorgiou, DDS, Dr Med Dent

Academic Editor

PLOS One

Additional Editor Comments (optional):

Reviewers' comments:

# Editor report: Acceptance letter

**DOI:** 10.1371/journal.pone.0352702.r004

**Authors:** Spyridon Papageorgiou, Spyridon Papageorgiou

PONE-D-25-63924R1

PLOS One

Dear Dr. Pauletto,

I'm pleased to inform you that your manuscript has been deemed suitable for publication in PLOS One. Congratulations! Your manuscript is now being handed over to our production team.

At this stage, our production department will prepare your paper for publication. This includes ensuring the following:

* All references, tables, and figures are properly cited

* All relevant supporting information is included in the manuscript submission,

* There are no issues that prevent the paper from being properly typeset

You will receive further instructions from the production team, including instructions on how to review your proof when it is ready. Please keep in mind that we are working through a large volume of accepted articles, so please give us a few days to review your paper and let you know the next and final steps.

Lastly, if your institution or institutions have a press office, please let them know about your upcoming paper now to help maximize its impact. If they'll be preparing press materials, please inform our press team within the next 48 hours. Your manuscript will remain under strict press embargo until 2 pm Eastern Time on the date of publication. For more information, please contact onepress@plos.org.

You will receive an invoice from PLOS for your publication fee after your manuscript has reached the completed accept phase. If you receive an email requesting payment before acceptance or for any other service, this may be a phishing scheme. Learn how to identify phishing emails and protect your accounts at https://explore.plos.org/phishing.

If we can help with anything else, please email us at customercare@plos.org.

Thank you for submitting your work to PLOS ONE and supporting open access.

Kind regards,

PLOS ONE Editorial Office Staff

on behalf of

Dr. Spyridon N. Papageorgiou

Academic Editor

PLOS One
